# Diagnostic value of serum amyloid A in differentiating the inflammatory disorders in wild birds

**DOI:** 10.3389/fvets.2024.1284113

**Published:** 2024-02-06

**Authors:** Haerin Rhim, Myeongsu Kim, Seulgi Gim, Jae-Ik Han

**Affiliations:** ^1^Laboratory of Wildlife Medicine, College of Veterinary Medicine, Jeonbuk National University, Iksan, Republic of Korea; ^2^Jeonbuk Wildlife Center, Jeonbuk National University, Iksan, Republic of Korea

**Keywords:** acute-phase protein, biomarker, birds, diagnostics marker, inflammation, serum amyloid A (SAA), wildlife

## Abstract

Rescued wild birds have very high rates of inflammatory diseases; however, there have been limitations in assessing them sensitively. Few studies have examined acute-phase proteins in wild birds. In this study, serum amyloid A (SAA) was evaluated as an inflammatory indicator along with traditional indices such as white blood cell count, albumin, and albumin/globulin ratio. In total, 291 samples from 139 birds of six avian species were analyzed. All samples were divided into four groups (severe, moderate, mild injuries, and clinically healthy) based on clinical examinations and evaluated by group. SAA levels were measured using an anti-chicken SAA ELISA kit and compared with leukocyte counts, albumin concentrations, and albumin/globulin ratios. Differences among groups were evaluated using the Kruskal–Wallis test, followed by a *post hoc* test using Dunn’s multiple comparisons with SPSS V27. Statistical significance was set at a value of *p* of <0.05. The median concentration and interquartile range (ng/mL) of SAA in each group were 78.10 ng/mL (50.71–109.30), 31.15 ng/mL (19.85–49.24), 9.68 ng/mL (4.71–22.06), and 3.10 ng/mL (1.50–6.00). We observed a significant difference in the mean levels between the groups (*p* < 0.001), with the same results observed across species. All four indices showed significant differences in mean values between the groups (*p* < 0.001). In addition, SAA showed rapid changes in periodically collected samples, reflecting either a positive treatment response or the onset of subclinical diseases. SAA can be used to detect inflammatory conditions and asymptomatic disease in wild birds and is helpful in accurately identifying current health status, which is essential for successful treatment and release.

## 1 Introduction

Traditionally, white blood cell (WBC) counts, including band neutrophil or heterophil counts, have been used as the first screening factor for inflammation, along with globulin levels and the albumin/globulin (A/G) ratio. Although the WBC count increases in many inflammatory responses, it can also increase in non-inflammatory cases, and even if it is decreased or within the normal range, inflammation cannot be excluded (1). In addition, after a temporary increase in leukocytes in the transient circulating blood, a true increase in leukocytes occurs a few days later (2–4). Therefore, these factors are insufficient to evaluate health status and detect subclinical diseases in real time.

When the body is stressed, acute-phase reactions occur as non-specific defensive responses and produce acute-phase proteins (APPs), mainly in the liver (5, 6). Studies have identified several positive APPs that increase in expression in response to physiological stress in many animals (5–7). Such APPs have been used as supplemental diagnostic markers to evaluate inflammatory reactions in animals (7). In particular, C-reactive protein (CRP) in dogs and serum amyloid A (SAA) in cats have been routinely tested in small animal clinics as measurement kits have been commercialized. Known avian APPs include SAA, alpha-1 acid glycoprotein, ceruloplasmin, transferrin, fibrinogen, and haptoglobin (8–15). Among these avian APPs, SAA has been the focus of research because it is a precursor of the amyloid A (AA) protein. However, a clear relationship has not been revealed between SAA and secondary amyloidosis, which is caused by the abnormal accumulation of AA in certain organs, although chronically elevated SAA concentrations are a prerequisite for the development of AA amyloidosis (16–19).

To date, methods that have been used to evaluate the inflammatory responses in birds are limited. Serum or plasma protein electrophoresis (SPEP) has been performed on avian plasma or serum to analyze serum protein and globulin fraction (20–25). Birds, mainly psittacine, with aspergillosis, chlamydiosis, sarcocystosis, nephritis, bacterial hepatitis, and mycobacteriosis, showed various commonly elevated globulins (26–29). However, many birds are much smaller than common companion animals, which makes it difficult to collect blood. Even after excluding size, there remain difficulties owing to the lack of either a non-commercialized test method as a point-of-care product or a reference database. Data have been accumulated from species that are frequently kept as pets, such as parrots and chickens. Additionally, it is impossible to quantify specific proteins that are known to be more sensitive to SPEP (30).

As confirmed in our previous study, approximately 80% of the birds admitted to the wildlife centers were having inflammation by trauma and/or infection. Accurate health screening is essential for providing proper treatment and pre-release evaluation, which leads to an increase in the number of released animals successfully. Therefore, we aimed to analyze whether SAA can be applied as an APP in some avian species and how it relates to traditional inflammatory markers. The objectives of the study were to measure SAA in six species of rescued wild birds and assess the correlation between SAA and WBC, albumin, and albumin/globulin ratio. Our hypothesis was SAA would be higher in birds with inflammation and reflect the current health condition sensitively than the traditional indices. Having a more sensitive indicator in addition to the conventional inflammatory indices currently used could help us assess a patient’s condition more quickly and provide adequate treatment in the future.

## 2 Materials and methods

### 2.1 Animals

A total of 291 samples from 139 birds of six species were referred to Jeonbuk Wildlife Center of the Republic of Korea, from January 2019 to February 2021: common kestrel (*Falco tinnunculus*, *n* = 54, CK), feral pigeon (*Columba livia domestica*, *n* = 66, FP), northern boobook (*Ninox japonica*, *n* = 84, NB), Eurasian eagle owl (*Bubo bubo kiautschensis*, *n* = 48, EEO), common buzzard (*Buteo*, *n* = 21, CB), and northern goshawk (*Accipiter gentilis*, *n* = 18, NG). Most birds were adults, and juveniles were also included. Sex and age information were not considered. Only birds who underwent clinical examinations and had at least one confirmed diagnosis were included in this study.

### 2.2 Sample collection

This study collected and analyzed the test results conducted during the medical treatment of avian patients visiting the wildlife center. A blood sample of 0.3–1 mL was obtained according to the bird’s species and condition from a basilic vein or a jugular vein. Most blood draws were performed with the bird’s face covered and manually restrained, unless they needed to be sedated for further examinations. The sample was collected in EDTA-containing tubes for CBC and sodium heparinized tubes for biochemistry panels during clinical examinations. A manual complete blood count (CBC) including blood cell counts, PCV, hemoglobin concentration, differential count, and blood smear evaluation was performed immediately following the reference method (31). Biochemical tests were conducted on the same day before separating the plasma. Biochemical panels and blood gas analyses were performed using VETSCAN VS2 (Zoetis, Parsippany, NJ, United States) and i-STAT (Abbott, East Windsor, NJ, United States). The plasma samples after measuring were stored at −20°C until SAA assays. All samples were subjected to at least one freeze–thaw cycle to measure SAA. In the case of multiple blood collections from one individual, each sample was considered separate because it was collected at intervals of 1 week or more. Some species were tested including banked samples frozen at −20°C between 2017 and 2018. Frozen samples were included in CK, NB, and EEO but accounted for less than 30% of the number of samples for each species.

### 2.3 Clinical groups

The samples were divided into four clinical groups based on the overall assessment of the bird’s condition: severe (S), moderate (M), mild (m) injuries, and clinically healthy (Ch). This classification was based on the type of injury diagnosed by physical examination, various diagnostic tests including CBC, biochemistry panels, radiographs, microbiological testing, cytology, molecular diagnostics, ultrasonography, computed tomography, magnetic resonance imaging, necropsy, and histopathology if the bird died, along with the time since being rescued at the point of inspection. Even if the bird was in a severe condition at admission, the sample was classified based on the condition of the bird on the day of collection. The S and M groups included patients with fractures, muscle and skin injuries, local or systemic infections, ocular injuries, and traumatic brain injury (TBI). The severity in the two groups was determined by the presence of severe wounds, systemic infection, or systemic inflammation found out by hematology, along with the duration of the problem. Acute fractures, large open wounds, and systemic inflammation caused by infection were always considered as the S group. Birds classified into the m group had mild dehydration, mild exhaustion, abrasion, or haemosporidial infection, or individuals improved over time. The Ch group included birds that had good vitality, appetite, and no clinical/laboratory abnormalities.

### 2.4 Follow-up evaluations

Moreover, in birds that underwent serial examinations, the initial levels of SAA and subsequent concentrations were evaluated according to the treatment progress. Except for regular checkups for healthy and long-term hospitalized individuals, 56 birds of six species were included as follows: CK = 9, FP = 14, NB = 14, EEO = 9, CB = 5, and NG = 5. These birds had various conditions, including fractures, tissue injury and necrosis, osteomyelitis, ocular injury, TBI, skin abscesses, luxation, arthritis, malnutrition, cachexia, bite or gunshot wounds, hepatitis, and bacterial or fungal pneumonia. All initial samples were classified as Group S or Group M.

### 2.5 Enzyme-linked immunosorbent assay

The concentration of SAA was measured using an anti-chicken SAA ELISA kit (Eagle Biosciences, Amherst, NH, United States) following the manufacturer’s instructions. This kit was validated with chicken samples during the R&D phase to prove its efficacy from the manufacturer. We applied this kit after preliminary tests to evaluate the reliability of the test in six species used in this study as per Kjelgaard-Hansen et al. (32). The samples from each species yielded consistent measurements across serial dilution ratios. All clinical samples were diluted in a ratio of 1:10 prior to measurement. Patients with severe hemolysis and lipemia were excluded from the study. All measurements were blinded from the sample information and performed in duplicate. Positive and negative controls were included.

### 2.6 Statistical analysis

As not all data were normally distributed by the Kolmogorov–Smirnov test, SAA concentrations, WBC counts, and A/G ratios according to the group were analyzed using the Kruskal–Wallis test. Dunn’s multiple comparison test was used for *post-hoc* comparisons of all pairs of groups. Albumin concentrations were analyzed by one-way ANOVA followed by Holm–Sidak multiple comparisons. The relationship of SAA with WBC count, albumin, and A/G ratio was evaluated using Spearman’s correlation coefficient. All *p*-values were set at 0.05. SPSS V27 (IBM SPSS, Armonk, NY, United States) and GraphPad Prism V9 (GraphPad Software, San Diego, CA, United States) were used for all the statistical analyses.

## 3 Results

### 3.1 Avian SAA concentrations by groups

The Chicken SAA Kit employed in this study demonstrated satisfactory reliability for measuring serum SAA across six avian species. Non-diluted samples showed non-reliable results, but the linear regression equation of the diluted samples did not show significant deviations. Both intra- and inter-assay variations were less than 10% (intra-assay: 3.59% [range 2.35~12.31%]; inter-assay: 7.43% [range 0.07~18.18%]). The lowest detection limit (mean + 2*SD), determined by the diluent included in the kit, was 0.072 ng/mL. Furthermore, the assay not only effectively differentiated clinically healthy and inflammatory birds but could be used for the changes in SAA concentration following inflammatory stimuli and various recovery phases.

**Figure 1:**
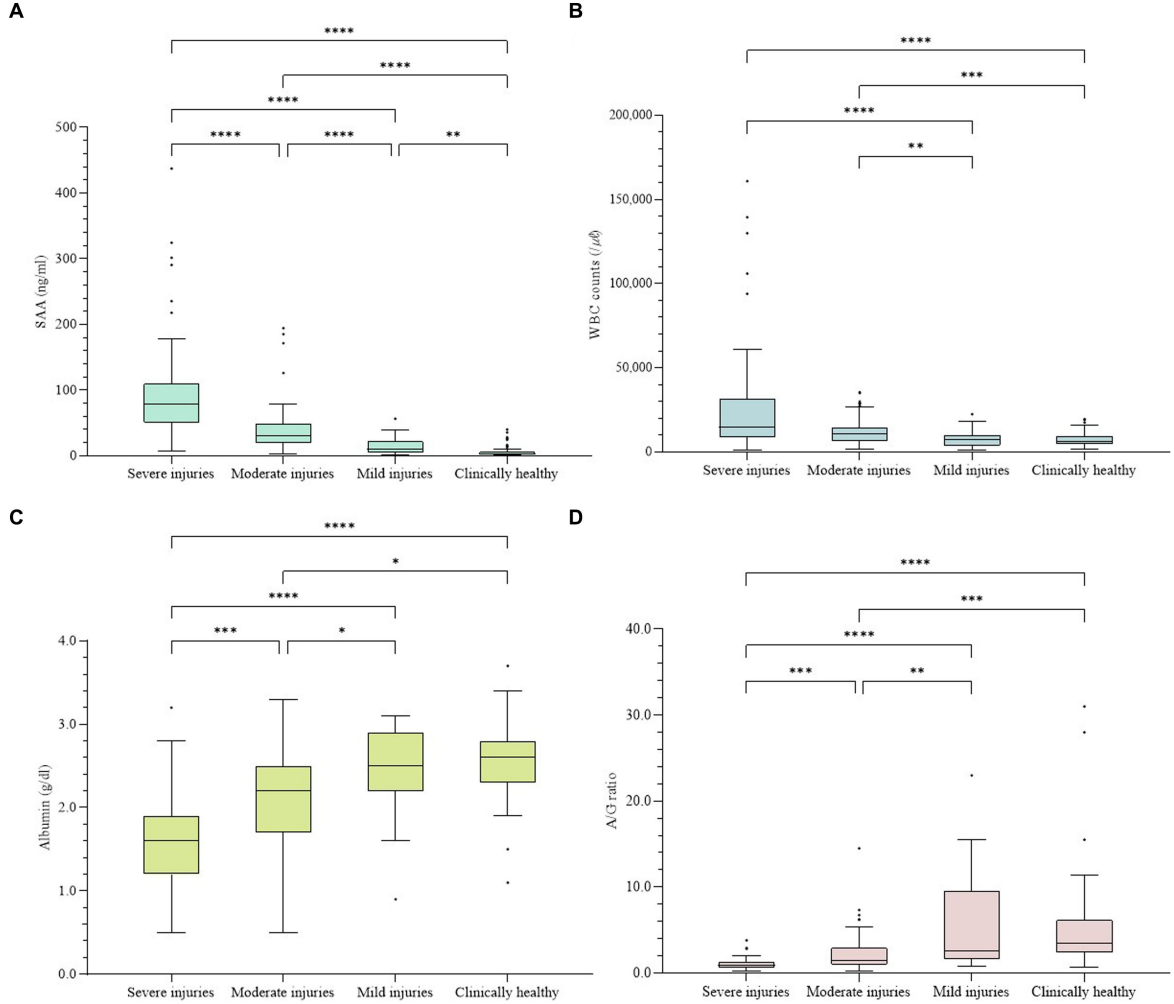
Concentrations of four indices in all samples. The difference was significant in every index among groups (*p* < 0.001). **(A)** Concentration of SAA. **(B)** WBC counts. **(C)** Concentrations of albumin. **(D)** A/G ratios. The plot includes whiskers (inner fence to outer fence), median (line within the box), 25th and 75th percentiles (box), and outliers (◆). Statistical significance was also confirmed for each of the two groups (*–****).

The SAA concentrations were analyzed for each group (Table 1; Figure 1). The measured SAA concentrations ranged from 0.8 ng/mL to 437.4 ng/mL. The median concentration (interquartile range) of group S was 78.10 ng/mL (50.71–109.30), group M was 31.15 ng/mL (19.85–49.24), group m was 9.68 ng/mL (4.71–22.06), and group Ch was 3.10 ng/mL (1.50–6.00). The more severe the disease classification, the higher the SAA level. When the SAA concentrations were compared for each clinical group, a significant difference was observed (*p* < 0.0001). Multiple comparisons also revealed statistically significant differences among all six cases in which the two groups were compared (*p* < 0.01).

**Table 1:** Data of SAA concentrations, WBC counts, albumin concentrations, and A/G ratio according to clinical groups.

Index	Species		S	M	m	Ch	Total	Value of *p*
SAA (ng/ml)	CK	No.	12	15	15	12	54	<0.0001
		Median	89.35	36.40	3.78	3.65		
		IQR	62.11–110.10	20.45–55.59	2.10–12.50	1.48–7.46		
	FP	No.	23	20	14	9	66	<0.0001
		Median	85.65	46.20	16.03	5.95		
		IQR	67.30–107.20	31.78–66.65	5.58–23.20	3.97–11.83		
	NB	No.	12	19	13	40	84	<0.0001
		Median	39.90	25.10	8.20	1.73		
		IQR	22.05–53.64	13.25–32.90	5.03–12.08	1.41–3.95		
	EEO	No.	12	20	11	5	48	0.0005
		Median	75.05	28.35	27.60	4.60		
		IQR	36.71–109.1	21.65–31.63	7.30–31.70	2.50–27.35		
	CB	No.	6	10	4	1	21	0.0025
		Median	85.58	39.90	7.38	5.00		
		IQR	69.69–299.00	26.51–91.89	4.63–16.28	5.00		
	NG	No.	4	9	5	0	18	0.0011
		Median	168.90	10.80	9.55			
		IQR	74.93–382.50	9.58–28.33	8.00–9.95			
	Total	No.	69	93	62	67	291	<0.0001
		Median	78.10	31.15	9.66	3.10		
		IQR	50.71–109.30	19.85–49.24	4.71–22.06	1.50–6.00		
WBC (/μL)	Total	No.	68	93	61	75	297	<0.0001
		Median	15,000	10,500	7,000	6,000		
		IQR	8,500–31,750	6,500–14,500	3,750–10,000	4,500–9,500		
Albumin (g/dl)	Total	No.	53	71	39	27	190	<0.0001
		Mean	1.59	2.08	2.44	2.53		
		95% CI	1.43–1.74	1.95–2.22	2.29–2.60	2.31–2.75		
A/G ratio	Total	No.	52	70	38	23	183	<0.0001
		Median	0.9	1.48	2.54	3.43		
		IQR	0.62–1.28	0.99–2.91	1.66–9.50	2.36–6.17		

The number, mean or median, 95% confidence level (CI) or interquartile range (IQR), and statistical value are shown. S, Severe injuries; M, moderate injuries; m, mild injuries; Ch, clinically healthy.

**Figure 2:**
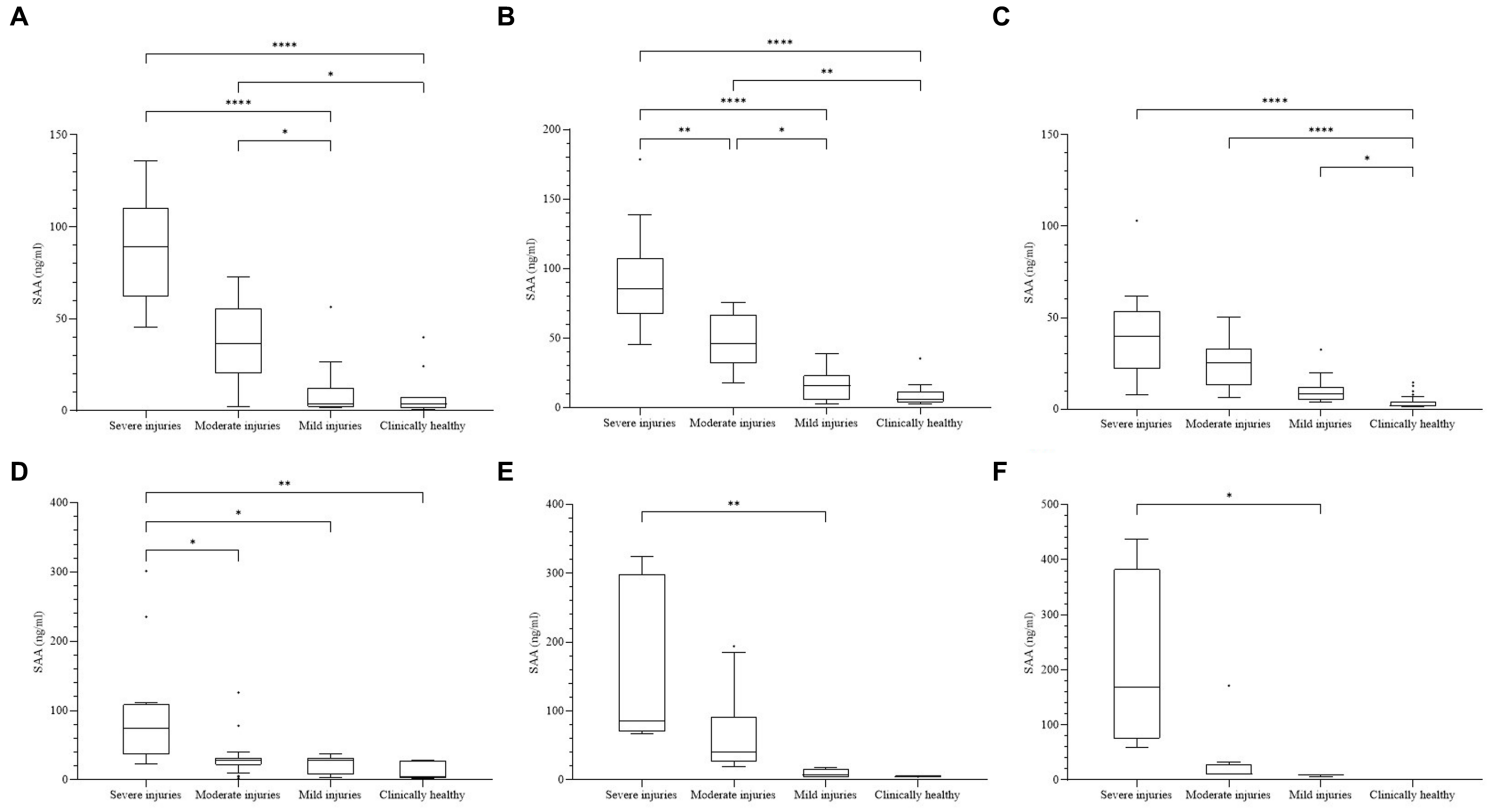
**(A)** Concentrations of SAA in each species. The difference was significant among all four clinical groups in every species (*p* < 0.01). **(A)** Common kestrel. **(B)** Feral pigeon. **(C)** Northern boobook. **(D)** Eurasian eagle owl. **(E)** Common buzzard. **(F)** Northern goshawk. Not a single bird was included in the clinically healthy group. The plot includes whiskers (inner fence to outer fence), median (line within the box), 25th and 75th percentiles (box), and outliers (◆). Significant differences between the two groups were indicated by the upper line and asterisk (*–****).

In the individual analyses of each species, the median concentration of SAA was significantly higher in the group with more severe problems for all six species, and significant differences were confirmed among the groups (Figure 2). In CK, significant differences (*p* < 0.0001) were observed between the four categories. The differences between the two groups were significant (*p* < 0.05), except for S-M and m-Ch. For FP, significant differences (*p* < 0.0001) were observed between the four categories. The differences between the two groups were significant (*p* < 0.05), except for m-Ch. For NB, significant differences (*p* < 0.0001) were observed among the four categories. The differences among S-Ch, M-Ch, and m-Ch were significant (*p* < 0.05). For the EEO, significant differences (*p* = 0.0005) were observed among the four categories. The difference was significant between S and the other three groups (*p* < 0.05). For the CB, significant differences (*p* = 0.0025) were observed among the four categories. The difference was significant only between the S and m groups (*p* = 0.0031). For NG, there were no samples from the Ch group, and the other three groups showed significant differences (*p* = 0.0011). The difference was significant only between the S and m groups (*p* = 0.0196).

Conditions in which a significant increase in SAA was confirmed included open fracture, closed fracture, osteomyelitis, tissue necrosis, skin laceration, bacterial or fungal pneumonia, TBI, ocular injuries, bacterial arthritis, bacterial hepatitis, skin abscesses, bite wounds, gunshot wounds, acute renal insufficiency, circulatory disturbance, and systemic or local bacterial infections. The levels outside the upper-outer fence consisted of individuals with severe exhaustion, lung abscesses, bacterial hepatitis, gunshot wounds, and ocular injuries accompanied by severe leukocytosis or leukopenia.

### 3.2 WBC, albumin, and A/G levels by groups

The data for other inflammatory indicators, including WBC count, albumin concentration, and A/G ratio, are shown in Table 1 and Figure 1. The WBC count ranged from 1,000 to 161,000 /μl, albumin ranged from 0.5 to 3.7 g/dL, and the albumin level ranged from 0.19 to 31.00. All three indices showed significant differences in comparing each group (*p* < 0.001). The more severe the classification level according to the disease, the higher the WBC count and the lower the albumin concentration and A/G ratio. When comparing the WBC counts of each group, multiple comparisons revealed statistically significant differences between the four cases: S-m, S-Ch, M-m, and M-Ch (*p* < 0.01). The comparison of albumin concentrations showed significant differences in all cases except m-Ch (*p* < 0.05). As a result of comparing the A/G ratio of each group, significant differences were found in all cases except m-Ch (*p* < 0.01).

### 3.3 Comparison of SAA with WBC, albumin, and A/G

Moderate correlations were found between SAA and albumin concentration (*r* = −0.476, *p* < 0.0001) and A/G ratio (*r* = −0.466, *p* < 0.0001). Additionally, a weak-to-moderate correlation was confirmed between SAA concentration and WBC count (*r* = 0.421, *p* < 0.0001) and PCV (*r* = −0.337, *p* < 0.0001). WBC counts showed a very weak relationship with albumin concentration (*r* = −0.177, *p* < 0.05) but a weak relationship with the A/G ratio (*r* = −0.266, *p* < 0.001) and PCV (*r* = −0.295, *p* < 0.001).

### 3.4 Follow-up evaluations

It was confirmed that the SAA level decreased with treatment in 44 of 56 birds and increased along with the inflammatory response in seven birds. The most common decreased cases were birds with elevated SAA due to fractures, which then decreased markedly as bones were stable, united, and inflammation subsided. Among the individuals whose SAA values were higher than the median level (31.15 ng/mL) of Group M, 20 birds showed a rapid decrease in the range of group Ch within 2 weeks. In two of the increased cases during hospitalization, this sudden reaction was attributed to the development of osteomyelitis, which decreased again following antibiotic treatment.

The three birds showed high levels for more than 3 weeks despite medication, matching clinical symptoms or other diagnostic tests that did not improve.

## 4 Discussion

While many studies on SAA have been conducted in common small and large animals, few studies have been conducted on birds. Immunoturbidimetry (TIA) or latex agglutination assay (LAS) for humans, ELISA kits, and TIA reagents for multiple species have been applied to various animals and evaluated by a reliable method (33–39). However, there was no cross-reactivity with samples from six species in our preliminary study using TIA reagent for human SAA (data not shown). It also has been reported that human SAA reagents have poor application in American flamingos (*Phoenicopterus ruber*) (40). Because birds include diverse species, the cross-reactivity of the SAA of each species and a chicken kit was unknown. Nevertheless, considering the use of methods with relative values rather than accurate levels for other species, meaningful measurement results were obtained for all six species in this study.

The SAA values were generally higher in the S and M groups, reflecting increased SAA in birds with inflammation mainly caused by trauma or infection. However, there was a large variation in the group. This could be explained by differences in individuals and ages or the reflection of disease severity. It has been confirmed in several species that SAA values are slightly higher at young ages (30, 41). Additionally, the value of APP varied according to the severity of the disease (42, 43). We created groups depending on the severity of the disease rather than the type, but time should also be considered. Unfortunately, not all blood samples were collected immediately after the incident because it can take hours for a wild bird to be transported. As APP increases from 3 to 6 h immediately after the incident, peaks at approximately 24 h, and then declines sharply, so it was difficult to determine where the bird was in the acute response (7). Although APP is known as not a specific indicator for specific diseases, the tendency to increase much more in cases of infection or trauma accompanying infection was confirmed in this study. Because birds were commonly rescued with diverse and combined conditions, the comparison of SAA for individual diseases was limited. Further studies are needed to determine how different SAA levels vary for specific conditions and by age in wild birds.

Approximately 9.3% (15/162) of the individuals in the S and M groups had near-normal values. Apart from the limitations of the current routine examination methods or individual differences, a disease that did not cause a marked increase in SAA or reduced liver function was suspected. It has been reported that if there is hepatic dysfunction in humans, APP production may be impaired due to decreased liver function, even in the presence of APR (44). A study in cats also found that liver disorders showed no significant increase in SAA, presumably due to a deteriorated liver (45). In the present study, there were eight samples with a low albumin level of 1.3 g/dL among the 10 cases with albumin results and low SAA values in the two inflammatory groups, and marked emaciation was confirmed in three of them. These cases are also consistent with the hypothesis that SAA synthesis of SAA was inhibited due to malnutrition or other primary causes of liver failure.

High SAA results were also found in 14% (18/129) of birds in groups m and Ch, which were expected to be low but were elevated. An alternative explanation is that these cases had a non-inflammatory reaction, and stress caused by rescue to a wildlife center or subclinical disease was considered. APP is also affected by age, diet, and non-inflammatory responses (13). However, no specimens exceeding 40 ng/mL were found in the clinically healthy or mild disease groups. Subclinical status was estimated in a study of cattle with high SAA but with no clinical symptoms, and abnormal SPEP results were found in 30% of clinically healthy parrots (46, 47). Because the production of APP can be affected by several parameters, serial follow-up inspections are recommended rather than a single test.

In 91% of birds with repeated follow-up measurements in one individual, it was confirmed that the SAA value decreased according to a positive response to the treatment process and the low SAA value increased significantly with the development of inflammation. It showed that SAA can be used as a diagnostic biomarker in birds. Rather than a single measurement, continuous monitoring is recommended to follow the trend. In approximately 40% of birds, SAA rapidly decreased to a normal range within 2 weeks, whereas in approximately 6% of cases, elevated SAA was observed from 3 to 8 weeks or more, and clinical symptoms did not improve, indicating that it accurately reflected their health status. They had bacterial arthritis or suspected viral infection. This study later revealed that SAA increased over time in one northern goshawk, in which only moderate intraocular hemorrhage, weakness, and anorexia due to blunt trauma were confirmed on the first basic physical examination. If SAA levels were confirmed during treatment, medication and additional tests would have been administered to relieve inflammation more quickly.

As not all organs have been thoroughly evaluated for amyloidosis in birds with chronically elevated SAA levels, we could not address the relativity of SAA and amyloidosis in our study. As not all organs were comprehensively assessed for amyloidosis in birds with persistently elevated SAA levels, our study could not explore the correlation between SAA and amyloidosis. Falcons have been reported to exhibit a significant increase in SAA even after 30 days of chronic disease, while manatees showed a return of SAA to the normal range after the same duration, emphasizing the need for follow-up studies in cases of prolonged inflammation (10, 35). Hampel et al. documented pathologically increased SAA in falcons with amyloid A amyloidosis (48). Nevertheless, some falcons with confirmed amyloidosis displayed low measurement values, leading the authors to speculate about decreased liver function at the final stage (10).

The WBC count is an index that is still mainly used with several factors to evaluate inflammation. Many studies have evaluated the association between SAA and WBC count in various animals, and the degree of association varies from poor to moderate, which was similar in this study (41, 49–53). They described that SAA outperforms WBCs by monitoring APR through discrepancies between WBC counts, including band neutrophil counts, and SAA levels. It was confirmed that APP is a more immediate indicator because SAA rapidly increased or decreased compared to WBC in this study. However, WBC count is assumed to affect the association analysis because it causes leukopenia in severe inflammatory reactions as well as leukocytosis. Of the 56 cases followed up, five birds with initially high SAA and leukopenia showed an elevation of WBC count when their condition was relieved, while SAA decreased. Although the WBC count was still high, seven patients showed a positive response to treatment and decreased SAA. An increase in SAA before an increase in the WBC count was observed in 11 individuals. Similar increases or decreases in both indices were observed in 26 animals. This meant that both markers were reliable inflammatory markers, but the fact that the test interval was at least 1 week did not sensitively reflect the increase or decrease in both markers should be considered. As mentioned in several studies, SAA and other hematological results can be applied in parallel to evaluate inflammation.

As described in many studies on birds, this study clearly confirmed a decrease in albumin and the A/G ratio in inflammatory diseases in all six species (14, 28, 29). The results by group show that the greater the severity, the more pronounced the decrease. Moreover, a moderate correlation with SAA was observed in both two indices. Therefore, when individual measurement of SAA is difficult, it could be beneficial to evaluate serial examinations of albumin and the A/G ratio. Still, there is a limitation in that we used albumin concentration measured using bromocresol green, which is not accurate for measuring albumin in birds and reptiles (54, 55).

Half of the samples in the present study were stored for more than 1 year. Although there have been no studies evaluating the stability of SAA in birds, it should be considered that its concentration might be affected by long-term storage. Many studies have been carried out based on the report that various APPs including human SAA remain stable when stored frozen at −20°C (56). On the other hand, frozen serum samples of cattle showed a significant decrease in SAA levels from the second day (57). Still, the data on the stability of SAA of birds in frozen samples are limited.

ELISA is suitable for testing many samples simultaneously and has the advantage that very few volumes are required; however, it requires precise manipulation and a long time. Additionally, it is expensive to inspect a small number of samples, and because of its high sensitivity, errors sometimes occur even for a skilled person. In the clinic, it is difficult to directly quantify or request protein analysis whenever needed. A specific APP or cytokine can be detected using PCR; however, this method cannot be used quickly in the medical field. If a kit or reagent capable of rapid diagnosis is developed in a point-of-care testing (POCT) setting, this test will be conducted more routinely in birds.

This is the first study that was conducted on many free-ranging birds admitted to a wildlife center to measure SAA and compare it with other inflammatory indicators. SAA showed high sensitivity as a biomarker of inflammation and could not only distinguish between the normal and inflammatory groups but also reflect their response over time, faster than changes in clinical symptoms or white blood cell counts, which will allow for a more meticulous evaluation of avian patients, which can help improve their welfare through accurate treatment and release decisions.

## Data availability statement

The original contributions presented in the study are included in the article/supplementary material; further inquiries can be directed to the corresponding author.

## Ethics statement

The animal study was approved by the Committee of Jeonbuk Wildlife Center. The study was conducted in accordance with the local legislation and institutional requirements.

## Author contributions

HR: Data curation, Formal analysis, Investigation, Methodology, Resources, Visualization, Writing – original draft. MK: Data curation, Investigation, Resources, Writing – review & editing. SG: Data curation, Investigation, Writing – review & editing. J-IH: Conceptualization, Formal analysis, Funding acquisition, Investigation, Methodology, Resources, Supervision, Validation, Writing – original draft, Writing – review & editing.
